# Fire Appliances for Use in Hospitals

**Published:** 1909-05-29

**Authors:** 


### HOSPITAL ADMINISTRATION.

### CONSTRUCTION AND ECONOMICS.

### Fire Appliances for Use in Hospitals.

#### I. CHEMICAL AND WATER EXTINGUISHERS.

With the view of fighting fire in its very earliest stages, a number of portable appliances have been worked out by different inventors and manufacturers. Portable appliances may properly be divided into two classes—those in which water only is employed, and those in which certain chemicals, bicarbonate of soda, sulphuric acid, and other substances are used to throttle the fire. There is a modification also of the latter form of apparatus, in which the chemicals are used in a dry powder, in place of being dissolved in water, as is the case in the majority of portable appliances.

Dry Powder Appliances.

The reason for the use of a dry powder, in place of water impregnated with chemicals, is one which applies particularly to hospitals, seeing that so many of them have adopted the electric light. Fires are sometimes caused by what are termed short circuits in the electric wires. Two wires, carrying current to an electric lamp, insulated from each other, are accidentally brought into contact by their insulation being damaged, or by its having perished, and the result is a powerful current through the small wires supplying the electric lamps, which ignites any woodwork in the neighbourhood and starts a somewhat dangerous fire.

When a portable extinguisher is employed to put out such a fire, if the appliance is one arranged to deliver a stream of water on to the fire, there is great danger, in certain cases, of the operator of the appliance receiving a very severe shock, sometimes even a fatal one. In many districts alternating currents of 200 volts are now employed, and many individuals have been killed by coming accidentally in contact with a conductor carrying an alternating current at that pressure. The stream of water directed on to such a conductor would connect it with the metal vessel holding the liquid, and thence with the operator.

Details of Powder Extinguishers.

The apparatus in which dry powder is employed is in the form of a cartridge. A long copper cylinder contains the powder, a cap being placed over the top, the cap having a loop by which the cartridge is suspended. The attachment of the cap to the cartridge is sufficiently strong to support its weight under ordinary conditions. In case of fire, however, instructions are given to seize the metal cylinder firmly with both hands, pull it down from the hook that is supporting it, wrench off the cap in so doing, and then to direct the powder quickly and sharply on to the seat of the fire. The powder contains chemicals which are inert as they lie in the cartridge, but when shaken violently on to the heated mass, combine and in so doing generate a quantity of gas which arrests combustion.

Chemical Jet Appliances.

There are a number of these on the market, all constructed on very much the same lines. There is an outer containing vessel of copper, with a certain quantity of water, having bicarbonate of soda in solution. There is also a vessel containing a certain quantity of sulphuric acid. Under ordinary circumstances the solution of bicarbonate of soda and the sulphuric acid are kept carefully separated. When a fire occurs the two are brought quickly into connection, the result being the generation of a large quantity of carbonic acid gas, and the creation of a pressure which forces out a stream of water charged with the gas and with the sulphate of soda, which are formed by the chemical reaction between the sulphuric acid and the bicarbonate.

The chemical jet extinguishers are manufactured in various forms, to be carried on the back by the aid of straps, to be carried in one hand by a handle, and in slightly larger form to be carried on wheels. In all cases a nozzle is provided, and a short length of hose, so arranged that the operator can easily and quickly direct the stream of charged water on to the fire.

The mixing of the sulphuric acid and bicarbonate of soda is accomplished in different ways. In one form the sulphuric acid is contained in a bottle, held in a cage near the top of the vessel, and a metal plunger is provided, upon which a smart blow is given with any convenient instrument, the bottle being broken, and its contents precipitated into the solution of bicarbonate of soda below. In another form, the containing vessel is inverted, and is provided with a substantial ring, standing on what is the top when the apparatus is not in use, and serving to support the whole vessel when in operation. In another form, syphons similar to those used for mineral waters have a sparklet attached which gives a stream of water charged with carbonic acid only. It is claimed that, in addition to the throttling action of the carbonic acid, the sulphate of soda coats the burning mass, and very largely assists in preventing further combustion.

Water Appliances.

The appliances in which water only is used consist of vessels of various forms, sometimes arranged to be carried by hand and sometimes on wheels, all of them fitted with a small pump and a nozzle and short piece of hose. In the hand forms, the foot holds the apparatus in place, while one hand directs the nozzle and the other works the pump. A useful addition to this, arranged by Messrs. Merryweather, is a small electric motor, which can take current from the electric lighting service by plugging a connection on the wall.

*(To be continued.)*

